# Multifocal photoacoustic microscopy using a single-element ultrasonic transducer through an ergodic relay

**DOI:** 10.1038/s41377-020-00372-x

**Published:** 2020-07-31

**Authors:** Yang Li, Terence T. W. Wong, Junhui Shi, Hsun-Chia Hsu, Lihong V. Wang

**Affiliations:** 1 Caltech Optical Imaging Laboratory, Andrew and Peggy Cherng Department of Medical Engineering, Department of Electrical Engineering, California Institute of Technology, 1200 East California Boulevard, Pasadena, CA 91125 USA; 2 Department of Biomedical Engineering, Washington University in St. Louis, Campus Box 1097, One Brookings Drive, St. Louis, MO 63130-4899 USA; 3 Present Address: Translational and Advanced Bioimaging Laboratory, Department of Chemical and Biological Engineering, Hong Kong University of Science and Technology, Hong Kong, China

**Keywords:** Imaging and sensing, Microscopy

## Abstract

Optical-resolution photoacoustic microscopy (OR-PAM) has demonstrated high-spatial-resolution imaging of optical absorption in biological tissue. To date, most OR-PAM systems rely on mechanical scanning with confocally aligned optical excitation and ultrasonic detection, limiting the wide-field imaging speed of these systems. Although several multifocal OR-PA (MFOR-PA) systems have attempted to address this limitation, they are hindered by the complex design in a constrained physical space. Here, we present a two-dimensional (2D) MFOR-PAM system that utilizes a 2D microlens array and an acoustic ergodic relay. Using a single-element ultrasonic transducer, this system can detect PA signals generated from 400 optical foci in parallel and then raster scan the optical foci patterns to form an MFOR-PAM image. This system improves the imaging resolution of an acoustic ergodic relay system from 220 to 13 μm and enables 400-folds shorter scanning time than that of a conventional OR-PAM system at the same resolution and laser repetition rate. We demonstrated the imaging ability of the system with both in vitro and in vivo experiments.

## Introduction

Optical-resolution photoacoustic microscopy (OR-PAM) has found broad applications in biomedical research for its superb ability to image rich optical absorption contrast in biological tissue^1,2^. To date, most OR-PAM systems rely on confocally aligned optical illumination and acoustic detection to mechanically scan an object to form an image. Therefore, the imaging speed is limited by the scanning speed^3,4^. Multifocal OR-PA computed tomography (MFOR-PACT) has been developed by utilizing a microlens array with multiple optical foci and an ultrasonic transducer array to detect PA signals^5,6^. However, previous MFOR-PACT systems are complex and costly due to the use of an ultrasonic array and the associated multi-channel data acquisition system.

**Fig. 1:**
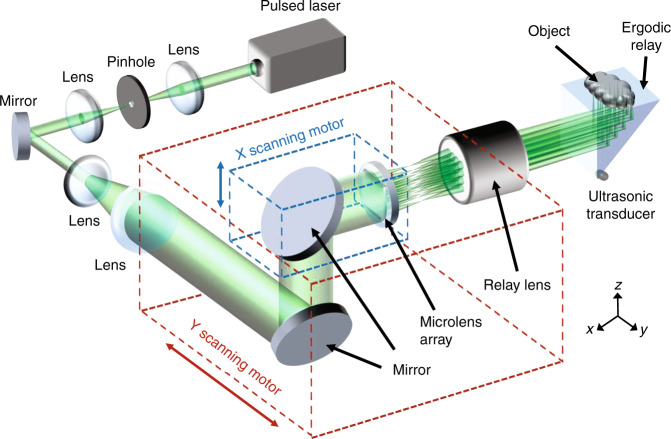
System schematic of MFOR-PAMER. A 532-nm pulsed laser beam is spatially filtered and expanded before being focused through a microlens array. The focusing plane of the microlens array is then imaged onto the imaging face of the ER prism to illuminate the imaging object. A single-element ultrasonic transducer is attached to a corner of the ER to detect the encoded PA signals

Here, we propose a two-dimensional (2D) MFOR-PAM system utilizing a 2D microlens array for optical excitation and an acoustic ergodic relay (ER) to simultaneously detect the PA responses to multifocal optical illumination with a single-element ultrasonic transducer (Fig. 1, Methods). We refer to this system as multifocal optical-resolution photoacoustic microscopy through an ergodic relay (MFOR-PAMER). The acoustic ER is ideally a low-loss acoustic propagation medium that allows input sound waves to be sufficiently scrambled inside the medium, resulting in distinct delay characteristics for each input position^7–9^. For PA imaging, an ER—such as a light-transparent prism—can be used as an encoder to transform PA signals from different input positions into unique temporal signals^10–12^. By recording the system impulse response of each input position in advance, the PA signals from the entire field-of-view (FOV) can be detected in parallel upon a single-laser shot. Then, the encoded PA signals can be decoded mathematically to reconstruct a 2D projection image of the object (see the ‘Methods’ section). Combining the powerful capability of an ER with a single-element ultrasonic transducer to detect multiple PA signals in parallel with a simple system setup, the MFOR-PAMER system is a low-cost alternative to a transducer array. In addition, the system uses a microlens array to focus a wide-field laser beam into multiple optical focal spots. Unlike a conventional focusing lens that needs to scan a single optical focal spot across the entire FOV, the microlens array can reduce the time required to form an image by scanning multiple optical focal spots altogether^14,15^. Since the excitation pattern through the microlens array is known, each optical focal spot can be computationally localized. By combining the microlens array with the ER, we can improve the acoustically defined image resolution (AR) of PAMER to the optically defined image resolution (OR) and shorten the scanning time by a factor equal to the number of microlens elements.

**Fig. 2:**
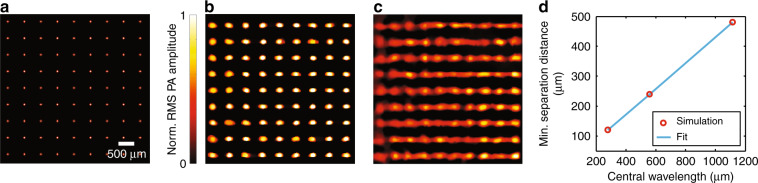
Simulation results of multifocal reconstruction. **a** Actual positions of the simulated microlens focal spots. **b** Reconstructed image of the simulated microlens array pattern when the pitch is acoustically resolvable. **c** Reconstructed image of the simulated pattern when the pitch is not acoustically resolvable. **d** Quantification of the minimum separation distance required to separate two spots in the MFOR-PAMER image at various transducer central wavelengths. Min., minimum; Norm., normalized

To demonstrate the capability of the MFOR-PAMER system, we first simulated the signals generated from a 2D microlens array with different detection parameters and then analysed the results (Methods). Figure 2a, b shows the actual positions of the simulated microlens focal spots with a pitch of 500 μm and the reconstructed image from a synthesized multifocal measurement, respectively. At this pitch, the PA signals from neighbouring optical foci are clearly separated. Each reconstructed spot is larger than the optical focal spot size because the system resolution is acoustically defined and related to the central wavelength of the detector^8,12^. Therefore, the reconstructed spots become smudged if the pitch is smaller than the system resolution, as shown in Fig. 2c. To quantify the relationship between the minimum separation pitch and the central wavelength, we replaced the 10 MHz transducer with other transducers with similar physical parameters but different central wavelengths. As Fig. 2d shows, the minimum separation pitch, defined as the distance between two focusing spots with a minimum contrast-to-noise ratio (CNR) of 6 dB, equals approximately 1/2 of the acoustic central wavelength in the ER.

**Fig. 3:**
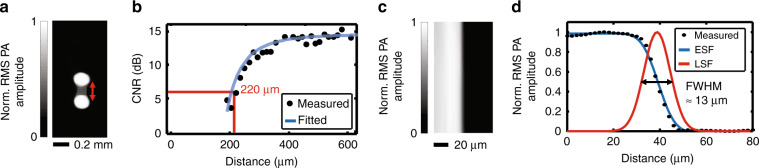
Quantification of the acoustic and optical lateral resolutions for MFOR-PAMER. **a** Quantification of the acoustic lateral resolution without localization. Two light spots shone on a black sheet were imaged to quantify the minimum distance needed to differentiate them in the reconstructed image. **b** Contrast-to-noise ratio (CNR) versus the distance between the two light spots. The lateral resolution, measured as the distance with a CNR of 6 dB, is 220 μm. **c** Quantification of the optical lateral resolution in MFOR-PAMER. An RMS PA amplitude projection image of a sharp edge is reconstructed by summing all the partial MFOR images. **d** Edge-spread function (ESF) used to calculate the line-spread function (LSF) of the system. The full width at half maximum of the LSF is ~13 µm. Norm., normalized

Without the microlens array, the MFOR-PAMER system is a conventional PAMER setup with an acoustically defined resolution^12,13^. In this article, we used two physical objects to quantify the image resolution with and without optical focal spot localization. To determine the AR without localization (Methods), we calculated the CNR versus the distance between the two laser spots, as shown in Fig. 3a. The lateral resolution, measured as the distance with a CNR of 6 dB in Fig. 3b, was 220 μm, which agrees well with the simulation results. To determine the OR using multifocal localization, we imaged the edge of a sharp metal blade (Methods). The reconstructed MFOR-PAMER image of the blade edge is shown in Fig. 3c. The averaged amplitude measurement was fitted to an error function to obtain the edge-spread function (ESF). We then calculated the line-spread function (LSF) of the system by taking the derivative of the ESF, as shown in Fig. 3d. The lateral resolution, measured as the full width at half maximum of the LSF, was 13 μm, matching the diameter of the focused laser spot.

**Fig. 4:**
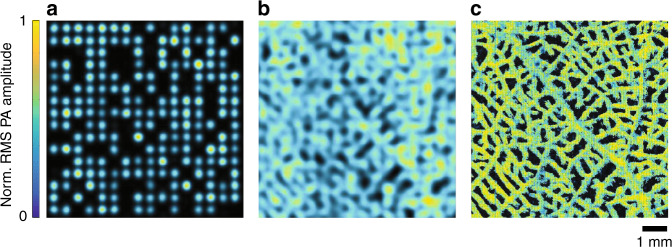
In vitro experimental images of a leaf skeleton vessel phantom. **a** Single-frame image of the microlens array excitation pattern. **b** AR-PAMER image from the direct summation of all reconstructed frames. **c** 2D MFOR-PAMER image obtained by localizing the optical foci from all scanning steps. Norm., normalized

We first demonstrated the performance of the system by imaging a leaf skeleton phantom, which contained a rich network of vessel-like structures, as shown in Fig. 4. A reconstructed image from a single-scanning step is shown in Fig. 4a. All the optical focal spots are well separated in the acoustically defined resolution pixels. The reconstructed images from all scanning steps were summed directly to create a conventional PAMER image without localization, as shown in Fig. 4b. This image is equivalent to an image obtained from wide-field light illumination. To enable a fair comparison, the summation procedure ensures identical total energy depositions and optical illumination profiles for the AR- and MFOR-PAMER images. The AR-PAMER image shows a blurry vessel skeleton in which many vessels cannot be visually separated and different vessel diameters cannot be differentiated. In contrast, the 2D MFOR-PAM image presents a detailed vascular skeletal network with different diameters and much finer image resolution, as shown in Fig. 4c.

**Fig. 5:**
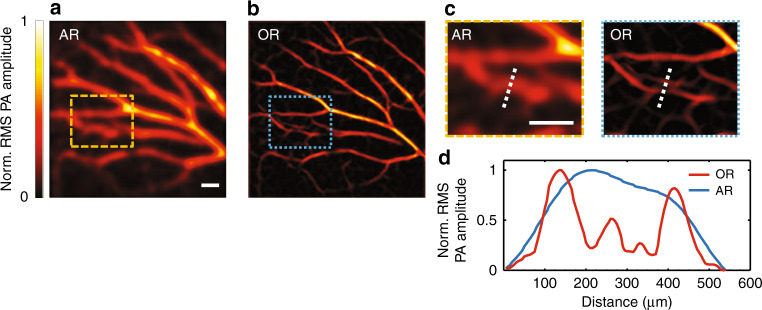
In vivo experimental images of the blood vasculature in a mouse ear. **a** AR-PAMER image. **b** MFOR-PAMER image. **c** Zoomed-in views of the boxed regions in (**a**) and (**b**). **d** Line profiles across the two white dashed lines in (**c**). AR, acoustic resolution; OR, optical resolution; Norm., normalized. All scale bars are 1 mm

Finally, we demonstrated the in vivo imaging ability of the MFOR-PAMER system by imaging blood vessels in a mouse ear. Figure 5a shows an AR-PAMER image of the ear vasculature. The vasculature appears blurry due to the poorer acoustically defined resolution. In contrast, the 2D MFOR-PAMER image in Fig. 5b shows a much-better-resolved vasculature. The zoomed-in views of the boxed regions from Fig. 5a, b and a comparison of the line profiles across the white dashed lines are shown in Fig. 5c, d. The MFOR-PAMER image reveals small vascular structures that can hardly be seen in the AR-PAMER image.

Many capillary-level vessels in the mouse ear cannot be resolved in the MFOR-PAMER image for two possible reasons. On the one hand, an optical resolution of 13 μm cannot fully resolve vessels that are much smaller in size. This limitation could be overcome by implementing a microlens array with a higher numerical aperture (NA) or by using a relay lens with a compression ratio to reduce the size of the optical focal spots. On the other hand, the current embodiment of the PAMER system suffers from low signal sensitivity due to signal attenuation inside the ER during propagation. As a result, signals from small vessels become too weak to be differentiated from background noise. This problem could potentially be solved by utilizing an ultrasonic detector with higher sensitivity and broader bandwidth, such as an optical micro-resonator detector^16^.

The current 2D MFOR-PAMER system forms an OR image with a 10 mm × 10 mm FOV and 2500 scanning steps in <10 s, excluding the training time. This system improves the imaging resolution of a PAMER system from 220 to 13 μm, a factor of ~17. Moreover, our system achieves a 400-fold improvement in scanning time compared with a conventional OR-PAM system at the same resolution and laser repetition rate by sacrificing the 3D spatial information to form a 2D projection image. Given a sufficient SNR, the training step can also be done within 10 s because only AR-quality training is required (Methods). These results significantly surpass those of previously demonstrated MFOR-PACT systems^5,6^. Our 2D MFOR-PAMER still has room for improvement. We expect that the image resolution, scanning time, and system sensitivity can be further optimized by utilizing an ultrasonic detector with higher sensitivity and broader bandwidth and a microlens array with a larger NA and smaller pitch (in accordance with the minimum AR separation distance). Moreover, the addition of a water tank helped reduce reconstruction degradation between the training and MFOR measurement steps. However, a training step still needs to be repeated for each experiment. A global uniform calibration would greatly simplify this process.

In this article, we described an MFOR-PAM system that utilizes an ER with a single-element ultrasonic transducer to detect multiple optical foci in parallel. This design can reduce the scanning time by at least 400-folds than conventional OR-PAM systems at the same imaging resolution. Our 2D MFOR-PAMER system has promising potential for many biomedical applications, such as utilizing ultra-violet (UV) illumination for a high-speed, label-free histological study of biological tissues^17^. This design can reduce the imaging time from several hours (with a conventional UV OR-PAM system) to less than a minute, significantly improving the efficiency of clinical histology and diagnostics.

## Methods

### System setup

A right-angle prism made of UV fused silica (PS611, Thorlabs, Inc.; 2203 kg/m^3^ density, 73.6 GPa Young’s modulus) was chosen as the acoustic ER^12^. A miniature ultrasonic transducer (XMS-310, Olympus, Inc.; 10 MHz central frequency, 2 mm element size) was placed at a corner of the prism to break the symmetry. A two-channel digitizer (ATS9350, AlazarTech, Inc.; sampling rate of up to 100 MS/s) recorded the encoded PA signals. A 532-nm wavelength pulsed laser (INNOSAB IS8II-E, Edgewave GmbH; 2-kHz pulse repetition rate and 5-ns pulse width) was used for optical excitation. The laser beam was filtered and expanded by a pinhole and two lenses.

Prior to the multifocal measurement, a training step was performed to quantify the impulse response for each pixel across the FOV. A focusing lens (LA1509, Thorlabs, Inc.; 25.4-mm diameter and 100-mm focal length) was used to focus the laser beam to a 5-μm spot. The laser radiant exposure (or fluence) was 50 mJ/cm^2^. Via the PA effect^18,19^, PA waves were generated by focused laser excitation and propagated through the ER. The focal diameter and pulse width of the laser beam were much narrower than the central wavelength and the reciprocal of the bandwidth of the ultrasonic transducer, respectively. Consequently, the PA wave input to the PAMER system could be approximated as a spatiotemporal delta function, and the detected signals quantified the impulse response of the linear system for the excitation position. The focused laser spot was raster scanned over the entire FOV—with a customized scanner consisting of two motorized translation stages (PLS-85, PI GmbH & Co.)—to record the point-by-point impulse responses.

After the training step, the focusing lens was replaced with a microlens array (64–479, Edmund Optics, 500-μm pitch, 1.2° divergence angle) to generate multiple optical foci. The working distance of a microlens array is relatively short, preventing us from focusing the optical foci onto the sample after passing through the ER. Therefore, a relay lens (272EN II, Tamron, 0.29 m minimum focus distance, 1:1 maximum magnification ratio) was used to increase the working distance while preserving the size of the optical focal spots. The laser radiant exposure at each optical focal spot was maintained at ≤20 mJ/cm^2^. PA signals generated from the multiple optical foci were then detected by the single-element ultrasonic transducer.

### Image reconstruction

The PA signals propagating through the ER can be expressed as a linear combination of the impulse responses from all the illuminated pixels:

1 $$s\left( t \right) = \mathop {\sum}\limits_{i = 1}^{N_p} {k_i} \left( t \right)P_i$$

where *s*(*t*) is the PA signal detected through the ER, *i* is the pixel index, *N*_*p*_ is the total number of pixels, *k*_*i*_(*t*) is the normalized impulse response, and *P*_*i*_ is the local PA amplitude at the *i*th pixel. Equation (1) can be recast in matrix form by discretizing time *t* according to the Nyquist criterion:

2 $${\mathbf{s}} = K{\mathbf{P}}$$

where $K = \left[ {k_1, \ldots ,k_{N_p}} \right]$ is the system matrix and **P** is the RMS PA amplitude image^12^. A two-step iterative shrinkage/thresholding (TwIST) algorithm^20^ was implemented to solve Eq. (2) for **P** as a minimizer of the objective function:

3 $$\widehat {\mathbf{P}} = {\arg} \,{\mathop {\min}\limits_{\mathbf{P}}}\left\| {{\mathbf{s}} - K{\mathbf{P}}} \right\|^2 + 2\lambda {\mathrm{{\Phi}}}_{{\mathrm{TV}}}({\mathbf{P}})$$

Here, Φ_TV_(**P**) is the total variation regularization term, and *λ* is the regularization parameter.

To generate a partial MFOR image, each reconstructed multifocal image was localized based on the true positions of the optical foci. The reconstructed images were digitally upsampled using the *imresize* function in MATLAB, the maximum amplitude of the pixels within each optical focal spot size (i.e., 13/2-μm radius around the localized centre position) was determined, and all pixels within the optical focal spot were set to that maximum value. Pixels outside the optical foci were zeroed out. Finally, all the partial MFOR images were summed to generate a 2D MFOR-PAMER image. During the multifocal measurement, only a distance equal to the pitch of the microlens array needs to be scanned to form a 2D MFOR-PAMER image. Therefore, the scanning time can be shortened by a factor equal to the number of microlens elements on the array to cover the same FOV.

### MFOR simulation with synthetic measurements

A training dataset of 500 × 500 pixels with a stepsize of 20 μm was first acquired by covering the imaging surface of the ER with black acrylic paint uniformly. To synthesize the multifocal measurement, we set *P*_*i*_ = 1 at the pixel positions where optical foci were generated by a simulated 2D microlens array and *P*_*i*_ = 0 at the other positions. The impulse responses from the training dataset for pixels under *P*_*i*_ = 1 were added up in the synthetic multifocal measurement. A zero-mean Gaussian random vector representing white noise was added to the synthesized signals. To quantify the relationship between the minimum separation pitch and the central wavelength, we used three ultrasonic transducers with similar physical parameters at central frequencies of 5 MHz (VP-0.5–5 MHz, CTS Electronics, Inc.; 5 MHz central frequency, 0.5 mm element size), 10 MHz (XMS-310, Olympus, Inc.; 10 MHz central frequency, 2 mm element size) and 20 MHz (VP-0.5–20 MHz, CTS Electronics, Inc.; 20 MHz central frequency, 0.5 mm element size).

### Quantification of resolution

For the measurement of the AR resolution, a training dataset of 100 × 50 points with a stepsize of 15 μm was first acquired by covering the imaging surface of the ER with a uniform layer of black acrylic paint (100× averaging, acquisition time = 5 min). Two 5-μm-diameter laser spots were simultaneously shone onto the black paint. While one beam was held stationary, the other beam was translated linearly away from the first during the PA measurements. For the measurement of the OR, a training dataset of 80 × 40 points with a stepsize of 2 μm was first acquired by covering the ER imaging surface with a thin metal blade painted with black acrylic paint uniformly (100× averaging, acquisition time = 4 min). After the training step, the metal blade was repositioned to have its edge resting in the middle of the FOV. A customized water tank was attached to the sample to reduce reconstruction degradation after the repositioning of the blade.

### In vitro imaging of leaf skeleton phantom

A piece of transparency film was cut to 25 mm × 25 mm and painted with black ink on one side for the training step. Since the imaged object is effectively a part of the ER system response, the leaf skeleton was attached to the film with ultrasonic gel to facilitate acoustic coupling. After the training step, the painted film was replaced with an unpainted film to image the leaf skeleton with the microlens array. A training dataset of 500 × 500 points with a stepsize of 20 μm was first acquired by covering the ER imaging surface with a thin film (~250 μm, similar to the thickness of the leaf skeleton phantom) painted with black acrylic paint uniformly (10× averaging, acquisition time = 30 min). After the training step, the thin film was replaced with the leaf skeleton phantom. A customized water tank was attached to the film and the phantom during the experiment to reduce reconstruction degradation after swapping the imaging samples. The microlens array was raster scanned with a 500 μm × 500 μm range and a stepsize of 20 μm to image the entire FOV.

### In vivo imaging of blood vessels in a mouse ear

Female ND4 Swiss Webster mice (Envigo; 18–20 g and 6–8 weeks) were used for the animal study. The laboratory animal protocols were approved by the Institutional Animal Care and Use Committee of the California Institute of Technology. The mouse was anaesthetized with 5% vaporized isoflurane mixed with air to induce anaesthesia and then transferred to a customized animal mount allowing the mouse ear to be laid flat on the imaging face of the ER. The mouse was anaesthetized with a continuous supply of 1.5% vaporized isoflurane during the experiment. A training dataset of 500 × 500 points with a stepsize of 20 μm was first acquired by covering the ER imaging surface with a thin film (~250 μm, similar to the thickness of the mouse ear) painted with black acrylic paint uniformly (10× averaging, acquisition time = 30 min). After the training step, the thin film was replaced with the mouse ear. A customized water tank was attached to the film and the mouse ear during the experiment to reduce reconstruction degradation after swapping the imaging samples. The surface optical fluence at each optical focal spot through the microlens array was maintained at ≤20 mJ/cm^2^ to comply with the ANSI safety limit per laser pulse^21^.

### Theoretical AR training

To satisfy the Nyquist criterion, the theoretical AR training stepsize needs to be <1/2 the AR imaging resolution (~100 μm for the current setup, as shown in Fig. 3). If we utilize the same laser at 2 kHz, the scanning stepsize will be ~70 μm to cover a 10 × 10 mm^2^ FOV in 10 s.

## Data Availability

The data that support the plots within this paper and other findings of this study are available from the corresponding author upon reasonable request and with permission from corporate collaborations.

## Code availability

The reconstruction algorithm and data processing methods are described in detail in the Methods section. We have opted not to make the computer codes publicly available due to corporate collaborations.
